# Smallholder Farmers’ Practices and African Indigenous Vegetables Affect Soil Microbial Biodiversity and Enzyme Activities in Lake Naivasha Basin, Kenya

**DOI:** 10.3390/biology10010044

**Published:** 2021-01-11

**Authors:** Eren Taskin, Chiara Misci, Francesca Bandini, Andrea Fiorini, Nic Pacini, Clifford Obiero, Daniel Ndaka Sila, Vincenzo Tabaglio, Edoardo Puglisi

**Affiliations:** 1 Dipartimento di Scienze e Tecnologie Alimentari per la Sostenibilità della Filiera Agro-Alimentare (DISTAS), Facoltà di Scienze Agrarie Alimentari ed Ambientali, Università Cattolica del Sacro Cuore, Via Emilia Parmense 84, 29122 Piacenza, Italy; eren.taskin@unicatt.it (E.T.); chiara.misci1@unicatt.it (C.M.); francesca.bandini@unicatt.it (F.B.); edoardo.puglisi@unicatt.it (E.P.); 2 Dipartimento di Scienze delle Produzioni Vegetali Sostenibili (DI.PRO.VE.S.), Facoltà di Scienze Agrarie Alimentari ed Ambientali, Università Cattolica del Sacro Cuore, Via Emilia Parmense 84, 29122 Piacenza, Italy; andrea.fiorini@unicatt.it; 3 Dipartimento di Ingegneria dell’Ambiente (DIAm), Università della Calabria, Via Pietro Bucci, 87036 Arcavacata di Rende, Italy; nic.pacini@unical.it; 4 School of Geography, Geology and Environment, University of Leicester, Leicester LE1 7RH, UK; 5 Land Resource Planning and Management, College of Agriculture and Natural Resources, Jomo Kenyatta University of Agriculture and Technology, Nairobi P.O. Box 62000, Kenya; cliffordobiero@jkuat.ac.ke; 6 School of Food and Nutritional Sciences, College of Agriculture and Natural Resources, Jomo Kenyatta University of Agriculture and Technology, Nairobi P.O. Box 62000, Kenya; dsila@jkuat.ac.ke

**Keywords:** soil biodiversity, soil bacteria, soil fungi, family farming, NGS, HTS, sustainable agriculture, farming practices, microbial diversity, soil enzymes, soil fertility, SSA

## Abstract

**Simple Summary:**

Smallholder farmers in Sub-Saharan Africa (SSA) are food insecure. Underexploited African indigenous vegetables (AIVs) are consumed locally without being considered a primary source of food and income. However, AIVs hold great potential for the future challenges of food security and climate change. We investigated the effects of different cropping systems and inclusion of AIVs in farming on the soil biodiversity and fertility status of smallholder farmers in Naivasha, Kenya. Compared to mainstream farming approaches, soil microorganisms under AIV cultivations differed significantly. Tillage, fertilization, soil amendments, and traditional homemade plant protection were singled out as the most important factors. The soil alteration index based on enzyme activity offered a reliable way to determine the alteration status for the first time in SSA. These findings could be useful for farmers to integrate AIVs with correct sustainable practices for a sustainable future and may contribute to the mitigation of food insecurity.

**Abstract:**

Loss of soil biodiversity and fertility in Sub-Saharan Africa (SSA) may put the food security of smallholder farmers in peril. Food systems in SSA are seeing the rise of African indigenous vegetables (AIVs) that are underexploited but locally consumed without being considered a primary source of food and income. Here we present a field study, a first of its kind, in which we investigated the effects of different cropping systems and inclusion of AIVs in the farming approach on bacterial and fungal biodiversity and community structures, enzymatic activity, and the alteration status of soils of the smallholder farmers in Kenya. When compared to mainstream farming approaches, the composition and biodiversity of bacteria and fungi under AIV cultivations was significantly different. Tillage had a significant impact only on the fungal communities. Fertilization and soil amendments caused shifts in microbial communities towards specialized degraders and revealed the introduction of specific microorganisms from amendments. Traditional homemade plant protection products did not cause any disturbance to either of soil bacteria or fungi. The soil alteration index based on enzyme activity successfully differentiated the alteration status for the first time in SSA. These findings could be useful for farmers to integrate AIVs with correct sustainable practices for a sustainable future.

## 1. Introduction

Food production and ecosystem services provision in Sub-Saharan Africa (SSA) are closely connected to soil fertility. For smallholder farmers, whose living conditions depend on the quality and quantity of resources obtained from agricultural land, soil fertility loss represents a particularly critical issue [1] by acting as a chronic poverty trap threatening their food security [2,3]. The next several decades are likely to witness a considerable rise in extreme events related to climate change and farmers from SSA are already facing the significant consequences [4,5,6]. Poor and unpredictable rains have recently led to diminished food production, with 2.7 million people threatened by food insecurity in Kenya alone and more than 200 million in countries across SSA [7,8,9]. In the last few decades, maize-based farming systems have had a critical role in supporting food security in SSA. Maize cultivation is usually followed by other cereals (millet, rice, and sorghum) in combinations with root and tuber crops where rain patterns are adequate [10]. However, smallholder farmers have yield gaps and severe production constraints in maize-based farming systems without the seeds of improved varieties of maize, adequate inputs, and soil fertility management practices [11]. The future of food systems, especially that of smallholder farmers in SSA, may benefit from the diffusion of African indigenous vegetables (AIVs), which are underexploited but often widespread and consumed in local communities without being considered staple crops [12,13]. AIVs (e.g., African nightshade (*Solanum scabrum* Mill.), spider plant (*Cleome gynandra* L.), and amaranth (*Amaranthus cruentus* L.)) are important constituents of the diets of over 60% of rural African communities, and they have the potential to prevent malnutrition, obesity, and diet-related disorders [14]. Leafy AIVs such as amaranth and spider plant also have the advantage of having a relatively short growth cycle (ready for harvest in a month or less) compared to staples such as maize [15,16] and were shown to be good candidate crops for stress conditions [17,18]. 

Recent findings indicate that the delicate balance of food security and agricultural sustainability hangs on a thin line between input intensification and careful management of agroecosystems [19,20]. Sustainable intensification of farms may be beneficial for the soil quality only when nutrient inputs and management practices are tailored according to soil properties, input availability, legume intercropping possibility, and irrigation [21,22,23]. In Kenya, soil fertility loss and erosion are major causes of the decline of agricultural land productivity [24]. Even in small amounts, improvements in farmers’ practices could improve the quality and quantity of yields obtained by smallholder farmers [25]. Tillage choice with integrated soil fertility management was found to be beneficial for soil arthropods diversity, infiltration rate, reduced surface run-off, and soil erosion mitigation [26,27,28,29,30,31]. However, implementation of such practices is not always possible for the time being. Especially in densely populated areas, a decline in total organic carbon, macro- and micronutrients, and soil organic matter is often expected when the land is converted from undisturbed to agricultural [32]. This could create the problem of soil mining that was often reported in smallholder farms with low input management systems in SSA [25,33].

Microbial biodiversity of soil is pivotal for the functionality of terrestrial ecosystems through the role of soil microbes in nutrient cycles and ecosystem services, especially under challenging conditions [34,35,36,37,38]. Despite its importance for the sustainability of farming systems, our knowledge on soil microbial diversity and its contribution to sustainability of smallholder farmers in SSA is currently very limited. This is especially relevant when considering that the majority of Kenyan farmers who cultivate AIVs, particularly those who are in remote rural areas, have only little to moderate adaptive capacity to climate change [39]. Soil microbial community structure and soil enzymes such as β-Glucosidase, phosphatase, and urease can act as early indicators of soil health and alteration because of their sensitivity to tillage, fertilization, cultivations, drought, pesticides, and pollution [40,41,42,43,44,45,46,47]. 

In Kenya so far, to the best of our knowledge, only Wanjiku Kamau et al. [48] investigated the impact of conventional and organic agriculture on soil fertility and biodiversity. Soil biodiversity in this study was investigated only through soil arthropods and it was found that farming practices were the key drivers of soil arthropod biodiversity and fertility, regardless of the location of the farms and biophysical conditions [48]. However, biodiversity of soil microbes is still a missing piece in a puzzle of soil fertility in smallholder farmers. Along a similar line of thought, our hypothesis was that the improved farming practices (tillage, fertilization, soil amendments, traditional homemade plant protection), together with AIV cultivation would also enhance soil fertility through soil microbial community, enzymes, and overall soil properties (porosity, aggregate stability, nutritive status, hydrological conditions, etc.).

The objectives of our study were to evaluate how smallholder farmers’ practices and inclusion of AIV affect soil microbial community and fertility in Lake Naivasha Basin, Kenya, through the assessment of:

- bacterial and fungal biodiversity;
- enzymatic activity; and
- soils alteration status when compared to mainstream farming approaches such as “maize monoculture” or “maize and beans.”

## 2. Materials and Methods

### 2.1. Site Description, Survey, and Soil Sampling

**Figure 1:**
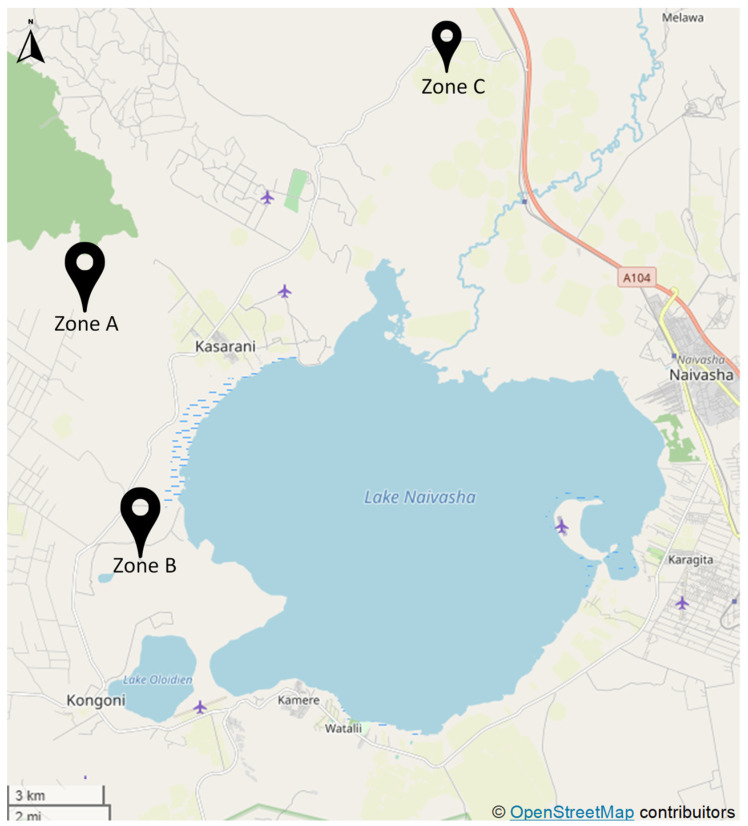
Farm locations around Lake Naivasha Basin, by zones (Creative Commons (CC) license by © OpenStreetMap contributors).

Fifteen fields managed by smallholder farmers were selected in the proximity of three locations, namely, Ndabibi (Zone A, 0°42′57.9″ S, 36°14′10.9″ E), Kongoni (Zone B, 0°45′50.5″ S, 36°16′05.6″ E), and Gilgil (Zone C, 0°26′59.0″ S, 36°16′19.0″ E) to survey all three within the Naivasha Basin in Nakuru County, Kenya (Figure 1). Sites surveyed in Zones A and B were previously classified by Jaetzold et al. [49] as ando-calcaric Regosols that are stratified, calcareous, well drained, loose fine sand to very friable sandy loam, or silt on lacustrine plains with moderate fertility. Zone C, on the other hand, was described as associated soils on the hills and minor scarps nearby with slightly higher fertility and lesser drainage [49]. 

The following specific criteria was used for the field selection together with the consent of farmers to participate in the study: Each zone needed to include at least one field where cultivation was maize based, at least one field where AIV were either directly cultivated or included in cropping/rotations, and finally, each zone needed to have various intensification levels in terms of farmers’ contributions to the alteration of the agroecosystem with agricultural practices such as tillage, continuous use of soil amendments, and agrochemicals. Selected fields were up to 1 ha in size to limit the variations in farm size [22]. All fields were located on one piece of land that were within a 100 m radius of the homestead, generally surrounded by natural vegetation. 

Through a questionnaire, information about farmers’ choices of cultivation/crop (maize and beans, vegetables, AIV: terere/kunde/saget/managu, AIV + vegetables), tillage (no-till, reduced, intensive), plant nutrition (compost + manure, compost + manure + urine, compost + manure + ash, commercial fertilizer, commercial fertilizer + manure) and plant protection products (none, homemade, commercial plant protection products) were gathered. General characteristics and management practices of surveyed farms according to the above variables are summarized in Table 1. 

Once the questionnaire was completed, fields were sampled following the guidelines detailed by ISO 18400-102 [50]. Three undisturbed soil samples (0–25 cm), from the corners of a 2.5 m equilateral triangle, were extracted by T-handle auger and combined as a composite sample in a sterile sample bag (Nasco Sampling/Whirl-Pak^®^, Madison, WI, USA). A total of four composite samples in each field were obtained (total of 12 extractions/field) and each composite soil sample was immediately placed in shade. The physico-chemical parameters of each surveyed field are provided in Supplementary Table S3. 

### 2.2. Analyses of Soil Microbial Diversity

The whole soil DNA was extracted using the DNeasy PowerSoil Kit (Ref 12888-100, QIAGEN GmbH, Hilden, Germany) according to the manufacturer’s protocol. Bacterial diversity was analyzed using the V3-V4 region of 16S ribosomal RNA (rRNA) gene and fungal diversity of the samples was analyzed by sequencing the Internal Transcribed Spacer 1 (ITS1) genomic region of ribosomal DNA (rDNA) as previously described in detail [37,51,52]. Thermal cycling conditions and information related to primers and reagents of PCR amplification are detailed in Supplementary Table S1. High-throughput sequencing data filtering, multiplexing, and preparation for concomitant statistical analyses were carried out as previously detailed [53,54]. Briefly, paired reads were completed with the “pandaseq” script [55] and sample demultiplexing was then carried out with the Fastx-toolkit (http://hannonlab.cshl.edu/fastx_toolkit/). Mothur v.1.32.1 [56] was used to remove sequences (i) with large homopolymers, (ii) that did not align within the targeted region, and (iii) that were chimeric [57]. The resulting sequences were analyzed with Mothur and R [58] by the operational taxonomic unit (OTU) and taxonomy-based approaches. For the former, the SILVA reference-aligned database for bacteria [59] was used with the NAST (Nearest Alignment Space Termination) algorithm and a kmer approach [60,61]. For the latter, sequences were classified into taxa using an amended version of the Greengenes database [62]. Sequence data were submitted to the National Center for Biotechnology Information (NCBI) Sequence Read Archive (SRA) BioProject ID PRJNA687992.

### 2.3. Soil Enzymatic Activity

β-Glucosidase (β-GLU, EC 3.2.1.21), phosphatase (PHO, E.C. 3.1.3.2), and urease (URE, E.C. 3.5.1.5) enzyme activity was determined on soil samples that were freshly sieved (<2 mm) then kept immediately at −20 °C until analysis. Assays to determine the activity of the abovementioned enzymes in soil samples were performed as previously described in detail by Eivazi and Tabatabai [63], Sannino and Gianfreda [64], and Kandeler and Gerber [65], respectively, and the slightly modified methods used in our study are detailed in Supplementary Table S2. The measured activity of these enzymes was then used to calculate the scores of Alteration Index 3 (AI3) by Puglisi et al. [47] with the following equation:

AI3=(7.87×βGLU) −(8.22×PHO) −(0.49×URE)

### 2.4. Statistical Analyses

Statistical analyses on OTU and taxonomy matrices were performed in Mothur and R and included hierarchical clustering with the average linkage algorithm at different taxonomic levels, principal component analysis (PCA) to assess the unconstrained samples grouping, and canonical correspondence analyses (CCA) to assess the significance of different treatments on the analyzed diversity. Metastats [66] was applied to identify features that were significantly different between management practices. Enzymatic activity and soil alteration index results were statistically analyzed by one-way analysis of variance (ANOVA) at a 95% confidence level. The means were statistically compared by the least significant differences (LSD) test using CoStat Statistical Software (Version 6400, CoHort Software Monterey, CA, USA).

## 3. Results

### 3.1. Analyses of Soil Microbial Diversity

Biodiversity of the bacterial and fungal communities in the surveyed fields were analyzed first through the total number of observed species (S_OBS_) and Chao’s and Simpson’s indexes (Table 2). The total number of observed species (S_OBS_) or species richness is literally the total number of species found in any given sample. 

The diversity indexes of Chao and Simpson, on the other hand, allowed us to estimate and compare the population diversity in the samples based on the abundances and evenness of the species in the samples, respectively [67]. In Zone A, Field 1, maize and beans were intercropped with rotations and continuous soil amendments had the greatest bacterial diversity and richness. Field 2, in which maize was cultivated as a monoculture without rotations, soil amendments had the greatest overall fungal diversity and richness. In Zone B, there were no significant differences between fields for either S_OBS_ or Simpson’s index for bacteria. According to Chao’s index, bacterial diversity in Fields 6 and 7 were highest. AIVs had either slightly but significantly lower or higher bacterial biodiversity than maize and beans intercropped in Field 5. Field 8 had the overall greatest fungal diversity and richness. Zone C, Field 10, where managu was cultivated intensively, had the greatest diversity and richness. Field 10 also had the overall greatest biodiversity and richness for fungi, which were lowest in SSN1 (Table 2). In addition, we analyzed the impact of farming practices on overall changes across the fields and found that all the management practices had a significant impact on bacterial S_OBS_. Soil amendments did not cause significant changes in Simpson’s diversity index, whereas crop and plant protection choices were insignificant for Chao’s diversity index for bacteria. All assessed farming practices had significant impact on fungal S_OBS_ and biodiversity indices (Table 2). 

**Figure 2:**
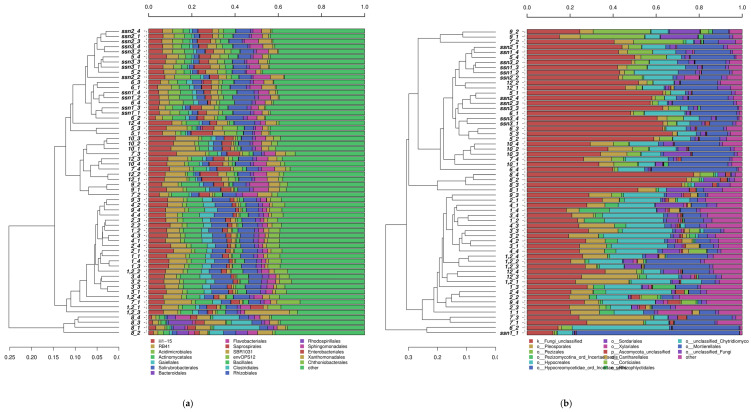
Taxonomic comparison of all samples ((**a**) bacteria, (**b**) fungi) through hierarchical clustering of microbial communities at the order level across all samples used in this study. Clusters were identified with the average linkage algorithm for taxa that contributed at least 5% to a single sample. Taxa that contributed less than this threshold were added to the sequence group denoted “other.”

The taxonomic comparison of all samples through hierarchical clustering of both fungal and bacterial communities at the order level across all samples used in this study is presented in Figure 2. Results indicate an intra-field and inter-field homogeneity of fields and zones, respectively. 

In soil bacterial communities (Figure 2a), the group of taxa that contributed less than a 5% threshold denominated as “other” was predominant in all samples. In Zone A (Fields 1, 1.2, 2, 3, and 4), predominance of “other” was followed by orders iii1-15, RB41, Actinomycetales and Solirubrobacterales, Rhizobiales, and Gaiellales. Zones B and C, except for Field 8, were clustered together. This cluster was further divided into two groups including Fields 5, 6, SSN1-2-3, and Field 7-9-10-12. In the former, predominance of “other” was generally followed by orders Rhodospirillales, Sphingomonodales, iii1-15, Saprospirales, and RB41. In the latter, it was followed by orders iii1-15, RB41, Sphingomonodales, Rhodospirillales, and Saprospirales. Field 8 was distinctively dominated by the Clostridiales, Bacteriodales, SBR1031, Rhizobiales, and Rhodospirillales orders (Figure 2a). 

Taxonomic comparisons of fungi revealed a clustering slightly more heterogeneous than bacteria. Zone A was clustered apart from Zones B and C with few exceptions. In the fields from Zone A (1, 1.2, 2, 3, and 4), predominance of unclassified fungi was generally followed by Hypocreales, Pleosporales, Mortierellales, and Pezizales. In the second group, in which most of the Zone B and C fields were clustered, with some exceptions, predominance of unclassified fungi was generally followed by Mortierellales, Hypocreales, Pezizales, Pleosporales, and Sordariales. Field 8 was distinctively dominated by unclassified fungi and predominance was followed by the Sordariales, Mortierellales, Pezizales, and Pleosporales orders (Figure 2b). 

**Figure 3:**
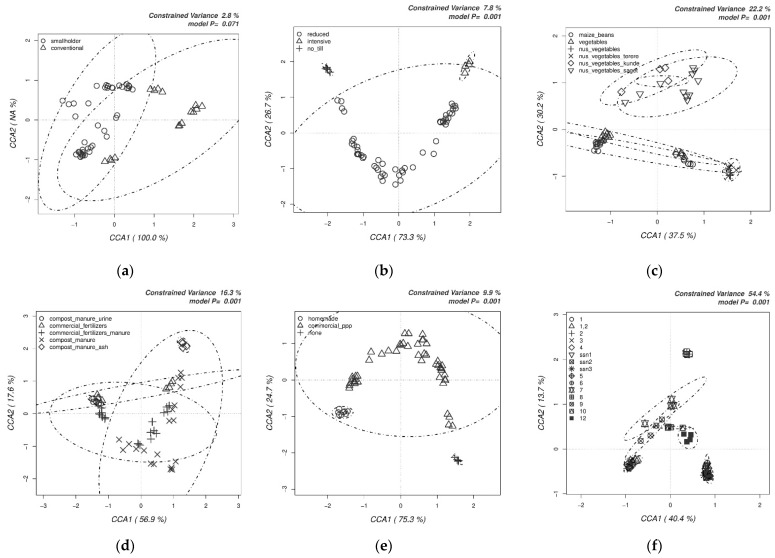
Canonical correspondence analyses (CCAs) on the impact of the farm management choices on the structure of bacterial communities. This was determined by the relative abundances of all the OTUs obtained by Illumina sequencing of bacterial 16S amplicons.

**Figure 4:**
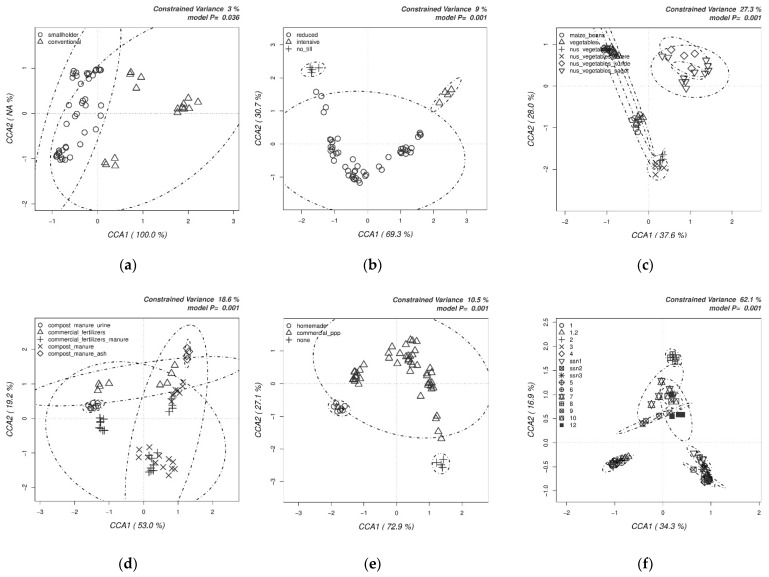
Canonical correspondence analyses (CCAs) on the impact of the farm management choices on the structure of fungal communities. This was determined by the relative abundances of all the OTUs obtained by Illumina sequencing of fungal ITS amplicons.

Bacterial and fungal OTU abundance tables were then subjected to multivariate canonical correspondence analysis (CCA) to assess the impact of various practices adopted by farmers on the structure of bacterial (Figure 3) and fungal communities (Figure 4). 

The farming approach, smallholder vs. conventional, did not significantly (*p* < 0.071, 2.8% variance) affect the clustering of the bacterial OTUs (Figure 3a). Neither reduced nor intensive tillage (Figure 3b) changed the clustering. However, no-till samples clustered significantly (*p* < 0.001, 7.8% variance). Crop choice was among the most important factors for the clustering of the bacterial communities (Figure 3c). Analysis revealed that AIVs clustered separately from common maize and beans and vegetables (*p* < 0.001, 22.2% variance). 

Similarly, fertilization choice also affected the clustering of bacterial communities, and amendment with ash or urine significantly contributed to the clustering of the bacterial communities (*p* < 0.001, 16.3% variance) (Figure 3d). Homemade plant protection products and the choice of not applying plant protection products clustered closely among themselves, whereas the use of commercially available plant protection products had wider impact on the clustering of bacterial communities of sampled farms (*p* < 0.001, 9.9% variance) (Figure 3e). Finally, some fields were clustered significantly close to each other depending on their zones, as expected, with slight exceptions (*p* < 0.001, 54.4% variance) (Figure 3f).

Clustering of the fungal OTUs was affected significantly (*p* < 0.05, 3% variance) by the farming approach adopted (Figure 4a). Tillage had a significant impact on the clustering of fungal communities (*p* < 0.001, 9% variance) (Figure 4b). Crop choice was also among the important factors for fungal communities (Figure 4c) as some AIVs clustered separately from common maize and beans and vegetables (*p* < 0.001, 27.4% variance). Fertilization choice with various amendments had a wide but significant impact on the clustering of fungal communities.

Amendment with ash or urine clustered separately. Inclusion of manure, either as mixed with commercial fertilizers or compost, formed bigger clusters than those with urine and ash (*p* < 0.001, 18.6% variance) (Figure 4d). Homemade plant protection products and those without any plant protection products clustered closely, whereas the use of commercially available plant protection products had a wider impact on the clustering of fungal communities of sampled farms (*p* < 0.001, 9.9% variance) (Figure 4e). Finally, fungal communities in the separate field were clustered significantly close to each other depending on their zone, as expected (*p* < 0.001, 62.1% variance). However, Fields 10 and 12 clustered close to Zone B, which was a different zone than their original one (Zone C) (Figure 4f).

### 3.2. Soil Enzymatic Activity

The measured activity of soil enzymes β-Glucosidase (βGLU), phosphatase (PHO), urease (URE), and the soil alteration index scores (AI3), which was calculated using the enzymatic activity of the fields, is reported in Table 3. 

Field 10 had the significantly highest alteration score (AI3 = 85.1) across all fields sampled in this study. Field 1.2 (AI3 = 42) and Field 9 (AI3 = 42.7) were the fields with highest alteration scores in their respective zones, A and B. In Zone A, Field 4 had the significantly lowest alteration score of AI = 6.08 (Table 3). In Zone B, the highest alteration score was that of Field 9 (AI3 = 42.61) and Field 5 was the lowest (AI3 = 16.96). In Zone C, where the highest scored Field 10 was located, SSN3 (AI3 = 30.8) was the lowest. The remaining fields resulted in scores that were not significantly different among themselves but significantly different from highest and lowest scored fields.

The results are summarized according to the significance of the impact that smallholder farmers’ treatments had on biodiversity, community structure clustering, and soil alteration in Table 4. The findings indicate that the differences in the surveyed fields were often the synergistic result of these variables together with the zones where the fields were located.

## 4. Discussion

Our findings indicate that a farming approach in which AIVs were rotated with maize and bean intercropping and coupled with moderate use of soil fertilization/amendment was found to be better at increasing soil biodiversity and soil status. This was due to fact that when compared to mainstream farming systems, the composition and biodiversity of bacteria and fungi under AIV cultivation was significantly different. These differences were mostly due to soil amendments/fertilization influence on soil microbial communities together with physico-chemical changes pointed out by the differences in enzyme activity in the fields.

### 4.1. Biodiversity of Bacterial and Fungal Communities

Crop choice, which is an important factor in soil microbial biodiversity [68], could explain the lower bacterial diversity in Zone A, where fields with vegetables had lower biodiversity compared to maize and beans. Agricultural practices and soil properties are influential on the microbial diversity in soils [69,70] and the farms in Zone B can explain the insignificant results obtained for the bacterial diversity in these fields due to similarity of the treatments and soil properties. In Zone C, even though Fields 10 and 12 were under intensive commercial production at the time of sampling, cases of a high number of observed fungal species may be attributed to the very recent conversion of these fields from natural areas to agricultural fields. This is because fungi play a crucial role in soil health, especially in the natural ecosystems, where they are often found in high numbers [71]. Actinomycetales contributes greatly to soil fertility via its role in the availability of nutrients and minerals, cycling of organic matter, and decomposition [72]. They are ubiquitous in soils and, as expected, they were present across all the samples in this study, thus confirming its presence in indigenous soil communities across the area. Their relatively higher abundance in Zone A, where all the farmers applied soil amendments, possibly stimulating the abundance of the bacterial taxa that play a role in abovementioned soil progresses, could be related to previous findings that indicated that Actinomycetales and Solirubrobacterales were found to be in their highest abundances in disturbed cultivation systems [73]. The slight difference observed in Zone A, besides its location, could be related to the application dose and frequency of the homemade soil amendments, as they are available throughout the year in the forms of animal manure, manure + urine, and compost in households where animals are kept. The presence and abundance of Rhizobiales in the sampled fields are of great importance since it is an associate of plants. Members of this order are well known for their plant growth-promoting traits and their provision of various nutrients, phytohormones, and precursors of plant metabolites to the plants [74,75]. Rhizobiales also includes members that are involved in the N cycling of soil; for short-term assimilation they perform the decomposition of the plant-derived materials in soil [76] and their abundances are also favored by long-term manure amendment [77]. All the fields sampled in this study received these amendments either directly as manure or indirectly through the inclusion of manure within compost that included recycled plant material. Changes in the relative abundances of Rhodospirillales and Sphingomonodales observed in our study were similar to those of Cao et al. [78] and can be explained by the differences in fertilization. This could be related to the fact that Rhodospirillales (as Rhizobiales) is a member of diazotrophic communities in soil, too, and together with Sphingomonodales could play a role in the degradation process of various soil amendments. The relative abundance increase observed in some of the maize and bean intercropping fields of Saprospirales is in accordance with the previous findings of De la Cruz-Barrón et al. [79] and similarly, may be related to the degradation of the maize residues applied to soil. Community composition at Field 8 was distinctive due to Bacteroidales, Clostridiales, and Flavobacteriales. Bacteroidales and Clostridiales are among the predominant microbial taxa of the fresh manures of the domestic sheep and cow. Therefore, the presence of these orders is in accordance with the fact that in these fields farmers often used manure and compost amended with fresh manure. It could also indicate the amendment of premature compost in the soils by the farmers. However, when manure is used within compost, the microbial community in the compost changes and Bacteroidales and Clostridiales usually leave their places to Actinomycetales, Sphingobacteriales, and Flavobacteriales by the end of the composting process [80,81]. The co-presence of these groups indicates a composting process that is not yet complete or the presence of fresh animal manure in the soils. Fields in Zones B and C differed from Zone A and Field 8 by the presence of Hypocreales and Sordariales. Hypocreales and Sordariales abundances could be favored by changes in temperature and drought conditions [82], and the presence of these two orders across the fields in our study can be explained by the sampling time, which was right before the long rainy season in Kenya. The Hypocreales order is a great source for entomopathogenic fungi that have proven useful for eradicating soil- and plant-borne pests [83]. The particularly high abundance in certain fields could be related to the inherent characteristics of those fields in terms of soil properties and management practices. Moreover, the Pezizales order was distinctive in Field 8, and these results were also similar to Ye et al. [84], in which fertilizer and manure application favored the Pezizales order. However, it should be noted that Ye et al. [84] highlighted a decrease in pH after fertilizer application as one of the factors. In our study, the pH of Field 8 was not significantly different than the fields sampled in the vicinity that received same treatments (Supplementary Table S3).

CCA analysis revealed that crop choice and fertilization were the two main drivers of change for both soil bacterial and fungal communities in Naivasha. The overall approach in our study, which was categorized as smallholder and conventional, was not significant due to the vast diversity of the specific practices used by farmers. These findings are in accordance with the previously well-established fact that crop choice and fertilization are among the most important factors affecting microbial diversity together with the overall management system as a whole [85,86,87,88]. The indifferent conventional and smallholder management approaches could be related to the intensification level of the farmers. Even in a conventional category, sampled farms were still being managed similarly with minimal or moderated external inputs that cannot be compared to conventional farming practices in European countries. The tillage choice of farmers was significantly important only for fungal communities of the sampled fields. This is in contrast with the previous findings that suggest that both bacterial and fungal communities were affected by physicochemical changes driven by tillage through soil homogenization and plant presence [89]. However, a contrasting study suggested that the tillage may have an impact on the distribution of both bacterial and fungal communities of the soil, depending on the vertical variation of the former and the relative abundances of the latter [90]. Our results are in accordance with their findings, suggesting that niche-based taxonomical differences in bacteria were more important for the impact of the tillage than fungi’s relative abundances in the sampled fields. The impact of the use of plant protection products that are homemade was found to limited. This is again related to the lack of know-how and correct preparation of homemade plant protection products and their applications in relatively low amounts. 

### 4.2. Enzymatic Activity

The activity of β-Glucosidase, phosphatase, and urease was found to be suitable for monitoring the alteration status of soils subject to different management [42,43]. Alteration Index 3 (AI3) [47] successfully integrated the activity of these enzymes to provide us with a comparative assessment tool for the evaluation of the impact of management practices on soil alteration status in our study. Higher scores for altered soils and lower scores for unaltered soils were assigned and AI3 index scores correctly indicated the highly altered status of Fields 1.2, 3, 7, 8, 10, and 12 in their respective zones. As expected, relatively lower alteration scores were often assigned to maize and bean fields. This is most likely due to the longer crop cycle of these crops, resulting in fewer interventions by farmers than shorter-cycle crops, as these enzymes are quite sensitive to temporal changes in management practices [91]. The most interesting case was Field SSN3 in Zone C, where a farmer managed his farm with an agroecological approach but also used commercially available pesticides and fertilizers. Field SSN3 scored significantly lower alteration scores than other fields in the same zone regardless of management approach. This contrasted with the alteration scores assigned to Fields 10 and 12, which can be attributed to heavier use of similar products and monocultures, as such practices over time can have a significant impact on the enzymatic activity of the soils [92]. The alteration index successfully indicated, in the short-term, possible physicochemical changes in the fields recently converted from natural areas to high intensity commercial AIV production as the most altered fields in this study in terms of ongoing soil fertility alteration. Furthermore, β-Glucosidase in particular, which is the most important enzyme for AI3, can be affected also by temporal and spatial differences occurring in these fields. These findings highlight the importance of an integrated approach to compensate for the possible side effects of intensification previously suggested by Pretty and Bharucha [93]. Similarly, in the African context, the AI3 index was previously used to evaluate the relationship between soil organic matter and tree performance in South Africa by Meyer et al. [94]. To the best of our knowledge, our study was the first time AI3 was applied to evaluate the soils in SSA, and in particular for smallholder farmers by proving it useful to evaluate the impact caused by the general management approaches of farmers on the sampled fields.

It must be also mentioned that farmers’ consent to sampling were great limitations on the selection of the fields due to cultural and sometimes linguistic barriers and inaccessible farm locations. This could explain the lack of similar studies in the literature for discussion and the lack of current interest from the research community to carry out these studies in a similar context. We also found that recordkeeping on these farms was seldom done, which is a great fallback in terms of data collection not only for further studies but also for anyone who would like to keep a record of the soil’s status. Without appropriate records, current and future impacts of farming practices—whether forward progress or a decline in resources provided by the soil—would be challenging to understand.

## 5. Conclusions

Our findings confirmed our hypothesis that farming practices together with AIVs have an impact on soil fertility through the soil microbial community, enzymes, and soil properties, as indicated especially by the results from the fields in which AIVs were integrated with maize and bean intercropping in rotations. When coupled with moderate use of soil fertilization/amendment, AIVs were found to be better at increasing soil biodiversity and soil status. In particular, Zone A provided a good example of how the management approach coupled with crop choice can positively affect bacterial and fungal diversity and richness. Zone B was as an example of how and to which extent changes can be driven by crop choice (AIVs) when they are subjected to the same management approach. Finally, Zone C provided hints about how an agroecological approach with the use of correct practices is relatively comparable to newly converted fields. It is plausible that some limitations due to the selection of fields may have influenced the results obtained and therefore, as might have been expected, pinpointing the reasons for each little difference in a field study of such scale is often unrealistic. However, despite all these hurdles, the present study still managed to contribute the knowledge about the impact of the current practices applied by local farmers on the soil microbial diversity while revealing possible associations between soil bacteria and fungi with AIVs.

## Figures and Tables

**Table 1:** General characteristics and management practices of surveyed fields (incl. = including, AIVs = African indigenous vegetables, C. fertilizer = Commercial fertilizer).

Zone	Field	Crop at the Time of Sampling	Rotated with	Management Approach	Tillage	Amendment(s) Fertilizer(s)	Plant Protection Products (PPP)	Irrigation
**A**	1	Maize & bean intercropping	Kunde (AIV), cabbage	Smallholder	Reduced	Compost, manure, urine	Homemade, compost tea	Rainfed
	1.2	Kale, broccoli	Onions, garlic, broccoli, kale, terere (AIV), carrot					Water harvest, Drip irrigation
	2	Maize and bean intercropping	Maize and bean intercropping	Conventional	Intensive	C. fertilizer	Commercial	Rainfed
	3	Cabbage, managu (nightshade—AIV)	Broccoli, kale, managu (AIV)	Smallholder	Reduced	C. fertilizer, manure		
	4	Maize and bean intercropping						
**B**	5	Maize and bean intercropping	Dill, nasturtium	Smallholder	Reduced	Compost	Commercial	Sprinkler from well
	6	Terere (amaranth—AIV)	Carrot, terere (AIV)			Compost, ash		
	7	Kunde (cowpea—AIV)	Cowpea, carrot, broccoli			Compost		
	8	Saget (spider plant—AIV)	Carrot, butternut squash					
	9	Managu (nightshade—AIV)	Previously empty					
**C**	SSN1	AIV mix	Cowpea, pigeon pea, chickpea, saget, sorghum	Smallholder	No till	Compost, manure	n/a	Sprinkler from well
	SSN2	Maize and bean intercropping	Cauliflower, beans	Conventional	Reduced	C. fertilizer	Commercial	
	SSN3	Terere, managu, pogo (AIV mix)	AIV mix, coriander, onions, cabbage, beans, carrot, melon, pumpkin			C. fertilizer, compost		
	10	Managu (nightshade)	Previously empty		Intensive	C. fertilizer, manure		Sprinkler from Melewa River
	12	Saget (spider plant)	Previously empty					

**Table 2:** Estimation of α-diversity indices and richness of each field separately for bacteria and fungi. The overall individual contribution of each practice on α-diversity is provided in the lower part (significance letters are formatted as a, *a*, A according to each zone and each diversity indicator. Different letters formatted in a similar manner indicate significant differences according to the one-way ANOVA carried out by LSD (Least Significant Differences) test at a 0.05 confidence. In the lower part of the table n.s.= not significantly contributing and + = significant contribution at *p* < 0.05).

		Bacteria			Fungi		
Zone	Field	S_OBS_	Simpson	Chao	S_OBS_	Simpson	Chao
**A**	1	3482 (±68) ^a^	492 (±22) ^a^	10439 (±483) ^a^	1835 (±48) ^b^	33 (±5) ^b^	3507 (±105) ^a^
	1.2	3288 (±95) ^b^	274 (±101) ^b^	9607 (±336) ^a^	2046 (±12) ^a^	32 (±6) ^b^	3735 (±302) ^a^
	2	3513 (±59) ^a^	428 (±16) ^ab^	10390 (±413) ^a^	2028 (±60) ^a^	59 (±17) ^a^	3878 (±110) ^a^
	3	3284 (±59) ^b^	403 (±21) ^ab^	9640 (±304) ^a^	1849 (±33) ^ab^	48 (±1) ^ab^	3727 (±129) ^a^
	4	3321 (±44) ^ab^	425 (±24) ^ab^	9534 (±388) ^a^	1825 (±48) ^b^	55 (±5) ^ab^	3414 (±101) ^a^
**B**	5	3114 (±64) *^a^*	410 (±20) *^a^*	8829 (±316) *^ab^*	1485 (±65) *^b^*	31 (±3) *^b^*	2692 (±88) *^b^*
	6	3173 (±70) *^a^*	349 (±26) *^a^*	9438 (±195) *^a^*	1144 (±114) *^bc^*	17 (±6) *^b^*	2078 (±212) *^c^*
	7	3202 (±50) *^a^*	365 (±16) *^a^*	9416 (±161) *^a^*	1443 (±44) *^c^*	22 (±2) *^b^*	2834 (±89) *^b^*
	8	3083 (±68) *^a^*	315 (±58) *^a^*	8735 (±104) *^b^*	1930 (±80) *^a^*	54 (±10) *^a^*	4319 (±244) *^a^*
	9	3051 (±37) *^a^*	366 (±25) *^a^*	8208 (±218) *^b^*	1620 (±155) *^b^*	27 (±9) *^b^*	3264 (±262) *^b^*
**C**	SSN1	2895 (±51) ^B^	299 (±15) ^C^	8867 (±233) ^CD^	1375 (±167) ^B^	16 (±7) ^B^	2728 (±190) ^C^
	SSN2	2842 (±39) ^B^	302 (±22) ^C^	8667 (±251) ^D^	1680 (±108) ^AB^	33 (±5) ^AB^	3098 (±183) ^BC^
	SSN3	3180 (±70) ^A^	325 (±26) ^BC^	9933 (±414) ^AB^	1522 (±84) ^AB^	33 (±3) ^AB^	2922 (±109) ^BC^
	10	3274 (±45) ^A^	415 (±19) ^A^	10597 (±368) ^A^	1822 (±36) ^A^	29 (±3) ^AB^	3415 (±141) ^AB^
	12	3244 (±35) ^A^	386 (±31) ^AB^	9652 (±156) ^BC^	1780 (±112) ^A^	38 (±9) ^A^	3728 (±235) ^A^
**Practices**	Crop	+	+	n.s.	+	+	+
	Tillage	+	+	+	+	+	+
	Fertilization	+	n.s.	+	+	+	+
	PPP	+	+	n.s.	+	+	+

**Table 3:** Measured enzyme activity of β-glucosidase (β GLU), phosphatase (PHO), and urease (URE) in fields, divided by zones (standard error of the means is indicated within parentheses); significance letters are formatted as a, *a*, A according to each zone and each diversity indicator; the letter after the parentheses indicates a statistical significance of *p* ≤ 0.05 by LSD test in ANOVA, by zones and enzymes. * Soil alteration is defined as in Puglisi et al. [47]).

		Soil Alteration *	Measured Soil Enzymes		
Zone	Field	AI3 Score	β GLU (μmol PNG g^−1^ h^−1^)	PHO (μmol PNP g^−1^ h^−1^)	URE (μg Urea g^−1^ h^−1^)
**A**	1	31.62 (±4.2) ^b^	9.99 (±0.6) ^a^	5.2 (±0.1) ^a^	8.8 (±0.4) ^a^
	1.2	41.94 (±2.6) ^a^	10.36 (±0.5) ^a^	4.44 (±0.2) ^b^	6.35 (±0.2) ^b^
	2	17.19 (±4.1) ^c^	5.94 (±0.8) ^b^	3.22 (±0.4) ^c^	6.28 (±0.4) ^b^
	3	37.82 (±3.7) ^ab^	10.04 (±0.5) ^a^	4.51 (±0.1) ^ab^	8.4 (±0.1) ^a^
	4	6.08 (±1.4) ^d^	3.83 (±0.2) ^c^	2.64 (±0.1) ^c^	4.71 (±0.1) ^c^
**B**	5	16.96 (±1.8) *^c^*	4.34 (±0.16) *^c^*	1.09 (±0.06) *^d^*	1.76 (±0.13) *^e^*
	6	27.34 (±4.2) *^b^*	6.09 (±0.72) *^c^*	0.5 (±0.06) *^e^*	2.45 (±0.08) *^d^*
	7	25.44 (±2.1) *^b^*	14.19 (±0.88) *^a^*	5.12 (±0.14) *^a^*	8.07 (±0.12) *^b^*
	8	24.33 (±1.5) *^b^*	12.43 (±0.98) *^a^*	3.74 (±0.1) *^c^*	5.18 (±0.12) *^c^*
	9	42.61 (±5.6) *^a^*	9 (±0.54) *^b^*	4.23 (±0.06) *^b^*	10.74 (±0.21) *^a^*
**C**	SSN1	65.56 (±6.9) ^B^	3.23 (±0.15) ^C^	0.73 (±0.16) ^C^	5.02 (±0.24) ^B^
	SSN2	64.59 (±8.2) ^B^	4.43 (±0.47) ^C^	0.72 (±0.06) ^C^	3.38 (±0.22) ^C^
	SSN3	30.76 (±4.2) ^C^	4.63 (±0.21) ^C^	1.18 (±0.1) ^B^	2.63 (±0.3) ^D^
	10	85.1 (±6.2) ^A^	13.21 (±0.74) ^A^	1.94 (±0.1) ^A^	5.95 (±0.1) ^A^
	12	63.47 (±5) ^B^	9.57 (±0.58) ^B^	1.1 (±0.1) ^B^	5.62 (±0.17) ^AB^

**Table 4:** Summary of the significance of the impact that practices have on biodiversity, community structure clustering, and soil alteration (+ indicates significance of at least *p* < 0.05, n.s.: not significant).

		Biodiversity						Community		Enzymes
		Bacteria			Fungi			Bacteria	Fungi	AI3 Score Impact
	Practice	S_OBS_	Chao	Simpson	S_OBS_	Chao	Simpson			
**Significance**	Crop	+	+	n.s.	+	+	+	+	+	+
	Tillage	+	+	+	+	+	+	+	+	+
	Fertilization	+	n.s.	+	+	+	+	+	+	+
	Plant protection	+	+	n.s.	+	+	+	+	+	+

## Data Availability

Sequence data were submitted to the National Center for Biotechnology Information (NCBI) Sequence Read Archive (SRA) BioProject ID PRJNA687992.

## Supplementary Materials

The following are available online at https://www.mdpi.com/2079-7737/10/1/44/s1. Table S1. PCR reaction mixtures and thermal profiles for different target genes used, Table S2. Detailed methodology used for enzymatic assays of soil samples, Table S3. Physicochemical parameters of each surveyed field.

## References

[1] Tully K., Sullivan C., Weil R., Sanchez P. (2015). The State of Soil Degradation in Sub-Saharan Africa: Baselines, Trajectories, and Solutions. Sustainability.

[2] Tittonell P., Giller K.E. (2013). When yield gaps are poverty traps: The paradigm of ecological intensification in African smallholder agriculture. Field Crop. Res..

[3] Mugo J.N., Karanja N.N., Gachene C.K., Dittert K., Nyawade S.O., Schulte-Geldermann E. (2020). Assessment of soil fertility and potato crop nutrient status in central and eastern highlands of Kenya. Sci. Rep..

[4] Knox J., Hess T., Daccache A., Wheeler T. (2012). Climate change impacts on crop productivity in Africa and South Asia. Environ. Res. Lett..

[5] Ringler C., Zhu T., Cai X., Koo J., Wang D. (2010). Climate Change Impacts on Food Security in Sub-Saharan Africa.

[6] Wolfram S., David B.L. (2010). Robust negative impacts of climate change on African agriculture. Environ. Res. Lett..

[7] (2017). Kenya and FAO: Partnering to Build Resilience and Food and Nutrition Security.

[8] FAOSTAT Kenya: FAO Country Profile. http://countrystat.org/home.aspx?c=KEN&p=co.

[9] FAO, IFAD, UNICEF, WFP, WHO (2020). Transforming Food Systems for Affordable Healthy Diets.

[10] Dixon J.A., Gibbon D.P., Gulliver A., Hall M. (2001). Farming Systems and Poverty: Improving Farmers’ Livelihoods in a Changing World.

[11] Droppelmann K.J., Snapp S.S., Waddington S.R. (2017). Sustainable intensification options for smallholder maize-based farming systems in sub-Saharan Africa. Food Secur..

[12] Campanaro A., Tommasi N., Guzzetti L., Galimberti A., Bruni I., Labra M. (2018). DNA barcoding to promote social awareness and identity of neglected, underutilized plant species having valuable nutritional properties. Food Res. Int..

[13] Baldermann S., Blagojević L., Frede K., Klopsch R., Neugart S., Neumann A., Ngwene B., Norkeweit J., Schröter D., Schröter A. (2016). Are Neglected Plants the Food for the Future?. Crit. Rev. Plant Sci..

[14] Pichop G.N., Abukutsa-Onyango M., Noorani A., Nono-Womdim R. (2016). Importance of indigenous food crops in tropical Africa: Case study. Acta Hortic.

[15] Ojiewo C., Tenkouano A., Hughes J.D.A., Keatinge J.D.H. (2013). Diversifying diets: Using indigenous vegetables to improve profitability, nutrition and health in Africa. Diversifying Food and Diets: Using Agricultural Biodiversity to Improve Nutrition and Health.

[16] Ojiewo C.O., Rubyogo J.C., Wesonga J.M., Bishaw Z., Gelalcha S.W., Abang M.M. (2018). Mainstreaming Efficient Legume Seed Systems in Eastern Africa: Challenges, Opportunities and Contributions Towards Improved Livelihoods.

[17] Guzzetti L., Fiorini A., Panzeri D., Tommasi N., Grassi F., Taskin E., Misci C., Puglisi E., Tabaglio V., Galimberti A. (2020). Sustainability Perspectives of *Vigna unguiculata* L. Walp. Cultivation under No Tillage and Water Stress Conditions. Plants.

[18] Taskin E., Boselli R., Fiorini A., Misci C., Ardenti F., Bandini F., Guzzetti L., Panzeri D., Tommasi N., Galimberti A. (2021). Combined Impact of No-Till and Cover Crops with or without Short-Term Water Stress as Revealed by Physicochemical and Microbiological Indicators. Biology.

[19] Mugizi F.M.P., Matsumoto T. (2020). Population pressure and soil quality in Sub-Saharan Africa: Panel evidence from Kenya. Land Use Policy.

[20] Pretty J., Toulmin C., Williams S. (2011). Sustainable intensification in African agriculture. Int. J. Agric. Sustain..

[21] von Arb C., Bünemann E.K., Schmalz H., Portmann M., Adamtey N., Musyoka M.W., Frossard E., Fliessbach A. (2020). Soil quality and phosphorus status after nine years of organic and conventional farming at two input levels in the Central Highlands of Kenya. Geoderma.

[22] Nyberg Y., Wetterlind J., Jonsson M., Öborn I. (2020). The role of trees and livestock in ecosystem service provision and farm priorities on smallholder farms in the Rift Valley, Kenya. Agric. Syst..

[23] De Mastro F., Traversa A., Brunetti G., Debiase G., Cocozza C., Nigro F. (2020). Soil culturable microorganisms as affected by different soil managements in a two year wheat-faba bean rotation. Appl. Soil Ecol..

[24] Mulinge W., Gicheru P., Murithi F., Maingi P., Kihiu E., Kirui O.K., Mirzabaev A., Nkonya E., Mirzabaev A., von Braun J. (2016). Economics of Land Degradation and Improvement in Kenya. Economics of Land Degradation and Improvement—A Global Assessment for Sustainable Development.

[25] Fischer S., Hilger T., Piepho H.P., Jordan I., Karungi J., Towett E., Shepherd K., Cadisch G. (2020). Soil and farm management effects on yield and nutrient concentrations of food crops in East Africa. Sci. Total Environ..

[26] Kihara J., Bolo P., Kinyua M., Nyawira S.S., Sommer R. (2020). Soil health and ecosystem services: Lessons from sub-Sahara Africa (SSA). Geoderma.

[27] Nyawade S.O., Gachene C.K.K., Karanja N.N., Gitari H.I., Schulte-Geldermann E., Parker M.L. (2019). Controlling soil erosion in smallholder potato farming systems using legume intercrops. Geoderma Reg..

[28] Njoroge S., Schut A.G.T., Giller K.E., Zingore S. (2019). Learning from the soil’s memory: Tailoring of fertilizer application based on past manure applications increases fertilizer use efficiency and crop productivity on Kenyan smallholder farms. Eur. J. Agron..

[29] Kiboi M.N., Ngetich K.F., Fliessbach A., Muriuki A., Mugendi D.N. (2019). Soil fertility inputs and tillage influence on maize crop performance and soil water content in the Central Highlands of Kenya. Agric. Water Manag..

[30] Jena P.R. (2019). Can minimum tillage enhance productivity? Evidence from smallholder farmers in Kenya. J. Clean. Prod..

[31] De Mastro F., Traversa A., Cocozza C., Pallara M., Brunetti G. (2020). Soil organic carbon stabilization: Influence of tillage on mineralogical and chemical parameters. Soil Syst..

[32] Willy D.K., Muyanga M., Mbuvi J., Jayne T. (2019). The effect of land use change on soil fertility parameters in densely populated areas of Kenya. Geoderma.

[33] Cobo J.G., Dercon G., Cadisch G. (2010). Nutrient balances in African land use systems across different spatial scales: A review of approaches, challenges and progress. Agric. Ecosyst. Environ..

[34] Baraniya D., Puglisi E., Ceccherini M.T., Pietramellara G., Giagnoni L., Arenella M., Nannipieri P., Renella G. (2016). Protease encoding microbial communities and protease activity of the rhizosphere and bulk soils of two maize lines with different N uptake efficiency. Soil Biol. Biochem..

[35] Delgado Baquerizo M., Maestre F.T., Reich P.B., Jeffries T.C., Gaitan J.J., Encinar D., Berdugo M., Campbell C.D., Singh B.K. (2016). Microbial diversity drives multifunctionality in terrestrial ecosystems. Nat. Commun..

[36] Jansson J.K., Hofmockel K.S. (2018). The soil microbiome-from metagenomics to metaphenomics. Curr. Opin. Microbiol..

[37] Vasileiadis S., Puglisi E., Arena M., Cappa F., Cocconcelli P.S., Trevisan M. (2012). Soil bacterial diversity screening using single 16S rRNA gene V regions coupled with multi-million read generating sequencing technologies. PLoS ONE.

[38] Nannipieri P., Ascher J., Ceccherini M.T., Landi L., Pietramellara G., Renella G. (2017). Microbial diversity and soil functions. Eur. J. Soil Sci..

[39] Chepkoech W., Mungai N.W., Stöber S., Lotze-Campen H. (2020). Understanding adaptive capacity of smallholder African indigenous vegetable farmers to climate change in Kenya. Clim. Risk Manag..

[40] Trivedi P., Delgado-Baquerizo M., Anderson I.C., Singh B.K. (2016). Response of Soil Properties and Microbial Communities to Agriculture: Implications for Primary Productivity and Soil Health Indicators. Front. Plant Sci..

[41] Schloter M., Nannipieri P., Sørensen S.J., van Elsas J.D. (2018). Microbial indicators for soil quality. Biol. Fertil. Soil.

[42] Nannipieri P., Trasar-Cepeda C., Dick R.P. (2018). Soil enzyme activity: A brief history and biochemistry as a basis for appropriate interpretations and meta-analysis. Biol. Fertil. Soil.

[43] Vischetti C., Casucci C., De Bernardi A., Monaci E., Tiano L., Marcheggiani F., Ciani M., Comitini F., Marini E., Taskin E. (2020). Sub-Lethal Effects of Pesticides on the DNA of Soil Organisms as Early Ecotoxicological Biomarkers. Front. Microbiol..

[44] Burns R.G., DeForest J.L., Marxsen J., Sinsabaugh R.L., Stromberger M.E., Wallenstein M.D., Weintraub M.N., Zoppini A. (2013). Soil enzymes in a changing environment: Current knowledge and future directions. Soil Biol. Biochem..

[45] Hartman K., Tringe S.G. (2019). Interactions between plants and soil shaping the root microbiome under abiotic stress. Biochem. J..

[46] Adetunji A.T., Lewu F.B., Mulidzi R., Ncube B. (2017). The biological activities of β-glucosidase, phosphatase and urease as soil quality indicators: A review. Soil Sci. Plant Nutr..

[47] Puglisi E., Del Re A.A.M., Rao M.A., Gianfreda L. (2006). Development and validation of numerical indexes integrating enzyme activities of soils. Soil Biol. Biochem..

[48] Wanjiku Kamau J., Biber-Freudenberger L., Lamers J.P.A., Stellmacher T., Borgemeister C. (2019). Soil fertility and biodiversity on organic and conventional smallholder farms in Kenya. Appl. Soil Ecol..

[49] Jaetzold R., Schmidt H., Hornetz B., Shisanya C., Agriculture M.O., Zusammenarbeit D.G.F.T. (2010). Farm Management Handbook of Kenya: Volume II: Natural Conditions and Farm Management Information.

[50] (2017). Soil Quality—Sampling—Part 102: Selection and Application of Sampling Techniques.

[51] Vasileiadis S., Puglisi E., Trevisan M., Scheckel K.G., Langdon K.A., McLaughlin M.J., Lombi E., Donner E. (2015). Changes in soil bacterial communities and diversity in response to long-term silver exposure. FEMS Microbiol. Ecol..

[52] Bandini F., Misci C., Taskin E., Cocconcelli P.S., Puglisi E. (2020). Biopolymers modulate microbial communities in municipal organic waste digestion. FEMS Microbiol. Ecol..

[53] Połka J., Rebecchi A., Pisacane V., Morelli L., Puglisi E. (2015). Bacterial diversity in typical Italian salami at different ripening stages as revealed by high-throughput sequencing of 16S rRNA amplicons. Food Microbiol..

[54] Vasileiadis S., Puglisi E., Arena M., Cappa F., Veen J.A., Cocconcelli P.S., Trevisan M. (2013). Soil microbial diversity patterns of a lowland spring environment. FEMS Microbiol. Ecol..

[55] Masella A.P., Bartram A.K., Truszkowski J.M., Brown D.G., Neufeld J.D. (2012). PANDAseq: Paired-end assembler for illumina sequences. BMC Bioinform..

[56] Schloss P.D., Westcott S.L., Ryabin T., Hall J.R., Hartmann M., Hollister E.B., Lesniewski R.A., Oakley B.B., Parks D.H., Robinson C.J. (2009). Introducing mothur: Open-source, platform-independent, community-supported software for describing and comparing microbial communities. Appl. Environ. Microbiol..

[57] Edgar R.C., Haas B.J., Clemente J.C., Quince C., Knight R. (2011). UCHIME improves sensitivity and speed of chimera detection. Bioinformatics.

[58] R Core Team (2012). R: A Language and Environment for Statistical Computing.

[59] Pruesse E., Quast C., Knittel K., Fuchs B.M., Ludwig W., Peplies J., Glöckner F.O. (2007). SILVA: A comprehensive online resource for quality checked and aligned ribosomal RNA sequence data compatible with ARB. Nucleic Acids Res..

[60] DeSantis T.Z., Hugenholtz P., Keller K., Brodie E.L., Larsen N., Piceno Y.M., Phan R., Andersen G.L. (2006). NAST: A multiple sequence alignment server for comparative analysis of 16S rRNA genes. Nucleic Acids Res..

[61] Schloss P.D. (2010). The effects of alignment quality, distance calculation method, sequence filtering, and region on the analysis of 16S rRNA gene-based studies. PLoS Comput. Biol..

[62] McDonald D., Price M.N., Goodrich J., Nawrocki E.P., DeSantis T.Z., Probst A., Andersen G.L., Knight R., Hugenholtz P. (2011). An improved Greengenes taxonomy with explicit ranks for ecological and evolutionary analyses of bacteria and archaea. ISME J..

[63] Eivazi F., Tabatabai M.A. (1990). Factors affecting glucosidase and galactosidase activities in soils. Soil Biol. Biochem..

[64] Sannino F., Gianfreda L. (2001). Pesticide influence on soil enzymatic activities. Chemosphere.

[65] Kandeler E., Gerber H. (1988). Short-term assay of soil urease activity using colorimetric determination of ammonium. Biol. Fertil. Soil.

[66] Paulson J.N., Pop M., Bravo H.C. (2011). Metastats: An improved statistical method for analysis of metagenomic data. Genome Biol..

[67] Kim B.R., Shin J., Guevarra R., Lee J.H., Kim D.W., Seol K.H., Lee J.H., Kim H.B., Isaacson R. (2017). Deciphering Diversity Indices for a Better Understanding of Microbial Communities. J. Microbiol. Biotechnol..

[68] Xiong C., Zhu Y.-G., Wang J.-T., Singh B., Han L.-L., Shen J.-P., Li P.-P., Wang G.-B., Wu C.-F., Ge A.-H. (2020). Host selection shapes crop microbiome assembly and network complexity. New Phytol..

[69] Wolińska A., Górniak D., Zielenkiewicz U., Goryluk-Salmonowicz A., Kuzniar A., Stȩpniewska Z., Błaszczyk M. (2017). Microbial biodiversity in arable soils is affected by agricultural practices. Int. Agrophys..

[70] Mhete M., Eze P.N., Rahube T.O., Akinyemi F.O. (2020). Soil properties influence bacterial abundance and diversity under different land-use regimes in semi-arid environments. Sci. Afr..

[71] Frąc M., Hannula S.E., Bełka M., Jędryczka M. (2018). Fungal Biodiversity and Their Role in Soil Health. Front. Microbiol..

[72] Bhatti A.A., Haq S., Bhat R.A. (2017). Actinomycetes benefaction role in soil and plant health. Microb. Pathog..

[73] Shange R.S., Ankumah R.O., Ibekwe A.M., Zabawa R., Dowd S.E. (2012). Distinct soil bacterial communities revealed under a diversely managed agroecosystem. PLoS ONE.

[74] Oleńska E., Małek W., Wójcik M., Swiecicka I., Thijs S., Vangronsveld J. (2020). Beneficial features of plant growth-promoting rhizobacteria for improving plant growth and health in challenging conditions: A methodical review. Sci. Total Environ..

[75] Sun L., Gao J., Huang T., Kendall J.R.A., Shen Q., Zhang R. (2015). Parental material and cultivation determine soil bacterial community structure and fertility. FEMS Microbiol. Ecol..

[76] Starke R., Kermer R., Ullmann-Zeunert L., Baldwin I.T., Seifert J., Bastida F., von Bergen M., Jehmlich N. (2016). Bacteria dominate the short-term assimilation of plant-derived N in soil. Soil Biol. Biochem..

[77] Lin Y., Ye G., Kuzyakov Y., Liu D., Fan J., Ding W. (2019). Long-term manure application increases soil organic matter and aggregation, and alters microbial community structure and keystone taxa. Soil Biol. Biochem..

[78] Cao Y., Zhou B., Wang X., Meng H., Zhang J., Li L., Hong J. (2020). Different fertilization treatments in coal mining-affected soils change bacterial populations and enable soil reclamation. Ann. Microbiol..

[79] De la Cruz-Barrón M., Cruz-Mendoza A., Navarro–Noya Y.E., Ruiz-Valdiviezo V.M., Ortíz-Gutiérrez D., Ramírez-Villanueva D.A., Luna-Guido M., Thierfelder C., Wall P.C., Verhulst N. (2017). The Bacterial Community Structure and Dynamics of Carbon and Nitrogen when Maize (Zea mays L.) and Its Neutral Detergent Fibre Were Added to Soil from Zimbabwe with Contrasting Management Practices. Microb. Ecol..

[80] Devane M., Robson B., Lin S., Scholes P., Wood D., Weaver L., Webster-Brown J., Gilpin B. (2020). Bacterial community shifts in decomposing cowpats and the subsequent impacts on fecal source indicators for water quality monitoring. Ecol. Indic..

[81] Mamun M.A.A., Sandeman M., Rayment P., Brook-Carter P., Scholes E., Kasinadhuni N., Piedrafita D., Greenhill A.R. (2020). The composition and stability of the faecal microbiota of Merino sheep. J. Appl. Microbiol..

[82] de Oliveira T.B., de Lucas R.C., Scarcella A.S.D.A., Contato A.G., Pasin T.M., Martinez C.A., Teixeira de Morales Polizeli M.D.L. (2020). Fungal communities differentially respond to warming and drought in tropical grassland soil. Mol. Ecol..

[83] Barnett K., Johnson S.N., Johnson S.N., Hiltpold I., Turlings T.C.J. (2013). Chapter One—Living in the Soil Matrix: Abiotic Factors Affecting Root Herbivores. Advances in Insect Physiology.

[84] Ye G., Lin Y., Luo J., Di H.J., Lindsey S., Liu D., Fan J., Ding W. (2020). Responses of soil fungal diversity and community composition to long-term fertilization: Field experiment in an acidic Ultisol and literature synthesis. Appl. Soil Ecol..

[85] Chen X., Henriksen T.M., Svensson K., Korsaeth A. (2020). Long-term effects of agricultural production systems on structure and function of the soil microbial community. Appl. Soil Ecol..

[86] Ishaq S.L., Seipel T., Yeoman C.J., Menalled F.D. (2020). Soil bacterial communities of wheat vary across the growing season and among dryland farming systems. Geoderma.

[87] Ouyang Y., Norton J.M. (2020). Short-term nitrogen fertilization affects microbial community composition and nitrogen mineralization functions in an agricultural soil. Appl. Environ. Microbiol..

[88] Schmidt J.E., Kent A.D., Brisson V.L., Gaudin A.C.M. (2019). Agricultural management and plant selection interactively affect rhizosphere microbial community structure and nitrogen cycling. Microbiome.

[89] Anderson C., Beare M., Buckley H.L., Lear G. (2017). Bacterial and fungal communities respond differently to varying tillage depth in agricultural soils. PeerJ.

[90] Sun R., Li W., Dong W., Tian Y., Hu C., Liu B. (2018). Tillage Changes Vertical Distribution of Soil Bacterial and Fungal Communities. Front. Microbiol..

[91] Miller M., Dick R.P. (1995). Thermal stability and activities of soil enzymes as influenced by crop rotations. Soil Biol. Biochem..

[92] Kwiatkowski C.A., Harasim E., Feledyn-Szewczyk B., Antonkiewicz J. (2020). Enzymatic Activity of Loess Soil in Organic and Conventional Farming Systems. Agriculture.

[93] Pretty J., Bharucha Z.P. (2014). Sustainable intensification in agricultural systems. Ann. Bot..

[94] Meyer A.H., Wooldridge J., Dames J.F. (2014). Relationship between soil alteration index three (AI3), soil organic matter and tree performance in a ‘Cripps Pink’/M7 apple orchard. S. Afr. J. Plant Soil.

