# Supplementary material for: Smallholder Farmers’ Practices and African Indigenous Vegetables Affect Soil Microbial Biodiversity and Enzyme Activities in Lake Naivasha Basin, Kenya
Source: Biology (Basel). 2021 Jan 11;10(1):44. doi: 10.3390/biology10010044 (PMC7826984; doi:10.3390/biology10010044)
Supplement: Supplementary file 1 [file biology-10-00044-s001.pdf]

biology

Supplementary material for the article

Smallholder farmers' practices and African Indigenous Vegetables affect soil microbial biodiversity and enzyme activities in Lake Naivasha Basin, Kenya

Eren Taskin[1], Chiara Misci[1], Francesca Bandini[1], Andrea Fiorini[2], Nic Pacini[3,4], Clifford Obiero[5], Daniel Sila[6], Vincenzo Tabaglio[2*] and Edoardo Puglisi[1]

* Correspondence: Prof. Vincenzo Tabaglio, vincenzo.tabaglio@unicatt.it; Tel.: +39-0523-599-222 (V.T.)

biology

Table S1. PCR reaction mixtures and thermal profiles for different target genes used

Target Gene	Reaction Mix	Volume (µL)	Step 1
16s rRNA<br>1st step	Phusion Flash High-Fidelity Master Mix	12.5	94 °C - 5 min<br>25x {94°C 30s; 50°C 30s; 72°C 30s}<br>72 °C 10 min.
	Nuclease free water	8	
	DNA template (1ng/µL)	2	
	Primer 343F (10µM)<br>(5′-TACGGRAGGCAGCAG-3′)	1.25	
	Primer 802R (10µM)<br>(5′-TACNVGGGTWTCTAATCC-3′)	1.25	
16s rRNA<br>2nd step	Phusion Flash High-Fidelity Master Mix	12.5	94 °C - 5min<br>10x {95°C 30s; 50°C 30s; 30°C 30s}<br>72 °C 10min.
	Nuclease free water	8	
	1st Step Amplicons	1.25	
	Primer 343F (10µM)<br>(5′-TACGGRAGGCAGCAG-3′)	1.25	
	Primer 802R (10µM)<br>(5′-TACNVGGGTWTCTAATCC-3′)	1.25	
ITS 1st step	Phusion Flash High-Fidelity Master Mix	12.5	94 °C - 4 min<br>28x {94°C 30s; 56°C 30s; 72°C 1min}<br>72 °C 7min
	Nuclease free water	8	
	DNA template (1ng/µL)	1.25	
	Primer ITS-1 (10µM)<br>(5'-TCCGTAGGTGAACCTGCGG-3')	1.25	
	Primer ITS-2 (10µM)<br>(5'-GCTGCGTTCTTCATCGATGC-3')	1.25	
ITS 2nd step	Phusion Flash High-Fidelity Master Mix	12.5	94 °C - 4 min<br>7x {94°C 30s; 56°C 30s; 72°C 1min}<br>72 °C 7min
	Nuclease free water	8	
	1st Step Amplicons	1.25	
	Primer ITS-1 (10µM)<br>(5'-TCCGTAGGTGAACCTGCGG-3')	1.25	
	Primer ITS-2 (10µM)<br>(5'-GCTGCGTTCTTCATCGATGC-3')	1.25	

PCR products generated from the second step were multiplexed as a single pool, separately for ITS and 16s amplicons and the pool was purified using Agencourt AMPure XP kit (REF A63880, Beckman Coulter, Milan, Italy) according to the manufacturer's protocol.

Table S2. Detailed methodology used for enzymatic assays of soil samples.

Enzyme	Incubated In	Incubation Conditions	Reaction Stopped Following Incubation by Adding	Activity Determined by
β-GLU	Buffered substrate solution (Modified Universal Buffer MUB pH 6.0 + 25mM 4-Nitrophenyl β-D-glucopyranoside)	Continuous shaking 250rpm, 37°C, 1h.	0.1M Tris-(hydroxylmethyl)-aminomethane pH 12 and 0.5M CaCl2 and vigorous shaking	Immediate separation of liquid phase and then spectrophotometrically at 405nm of liquid phase
PHO	Buffered substrate solution (MUB pH 6.5 + 25 mM p-nitrophenyl phosphatase)	Continuous shaking 250rpm, 37°C, 1h.	0.5M NaOH and 0.5M CaCl2 and vigorous shaking	
URE	Buffered substrate solution (Boric Buffer, pH 10 + 0.72M UREA)	Continuous shaking 250rpm, 37°C, 2h.	1N KCl/0.01N HCl solution and vigorous shaking	Liquid phase separated, then mixed with Sodium Salicylate solution (equal mix of 0.12% $Na_2Fe(CN)_5NO$, of 17% $C_7H_5NaO_3$ and H_20BD) and 1mL of 0.1% $C_3Cl_2N_3NaO_3$. Re-incubated at 24°C, 30 min and then spectrophotometrically determined at 690nm

Table S3. Physicochemical parameters of each surveyed field.

	TOC (%)	N (%)	P_{act} (mg/kg)	$K+_{act}$ (meq/100g)	pH (H20)	pH (KCl)	CEC (cmol /kg)	EC (ds/m)
F1	2.66 *(±0.19)**	0.23 *(±0.01)*	131 *(±13.86)*	2.37 *(±0.12)*	6.4 *(±0.19)*	5.52 *(±0.11)*	8.9 *(±0.4)*	0.08 *(±0.03)*
F1.2	2.06 *(±0.15)*	0.18 *(±0.01)*	100.08 *(±8.27)*	3.38 *(±0.12)*	6.6 *(±0.15)*	5.55 *(±0.05)*	8.3 *(±0.8)*	0.1 *(±0.01)*
F2	1.8 *(±0.13)*	0.16 *(±0.01)*	77.84 *(±2.28)*	3.63 *(±0.28)*	6.8 *(±0.05)*	5.37 *(±0.08)*	7.3 *(±0.5)*	0.05 *(±0.01)*
F3	2.36 *(±0.13)*	0.19 *(±0.01)*	101.47 *(±9.73)*	4.49 *(±0.17)*	6.5 *(±0.04)*	5.45 *(±0.03)*	6 *(±0.7)*	0.1 *(±0)*
F4	1.56 *(±0.03)*	0.13 *(±0)*	82.35 *(±1.59)*	4.65 *(±0.3)*	6.6 *(±0.06)*	5.13 *(±0.02)*	8.5 *(±0.5)*	0.08 *(±0)*
F5	1.43 *(±0.14)*	0.15 *(±0)*	288.07 *(±29.8)*	7.53 *(±0.28)*	8.7 *(±0.05)*	7.31 *(±0.06)*	5.6 *(±0.6)*	0.17 *(±0.01)*
F6	2.55 *(±1.09)*	0.17 *(±0.02)*	142.82 *(±20.51)*	8.7 *(±0.19)*	8.9 *(±0.06)*	7.48 *(±0.09)*	7.4 *(±1.1)*	0.19 *(±0.02)*
F7	1.67 *(±0.2)*	0.17 *(±0.01)*	137.95 *(±11.57)*	9.52 *(±0.31)*	8.7 *(±0.06)*	7.37 *(±0.05)*	11.9 *(±0.4)*	0.28 *(±0.02)*
F8	2.11 *(±0.22)*	0.22 *(±0)*	161.93 *(±20.25)*	11.23 *(±0.69)*	8.7 *(±0.07)*	7.28 *(±0.03)*	11.5 *(±2.6)*	0.33 *(±0.03)*
F9	1.16 *(±0.08)*	0.11 *(±0.01)*	283.9 *(±5.85)*	8.21 *(±0.29)*	8.9 *(±0.09)*	7.75 *(±0.04)*	12.5 *(±0.3)*	0.2 *(±0.03)*
F10	3.29 *(±0.21)*	0.21 *(±0.02)*	87.91 *(±6.26)*	3.97 *(±0.54)*	7.0 *(±0.21)*	6.28 *(±0.31)*	15.7 *(±1.4)*	0.6 *(±0.11)*
F12	4.76 *(±0.38)*	0.32 *(±0.02)*	268.95 *(±41.91)*	17.8 *(±3.32)*	7.6 *(±0.12)*	7.13 *(±0.11)*	10.4 *(±2.1)*	0.93 *(±0.18)*
SSN1	2.07 *(±0.17)*	0.12 *(±0.01)*	141.77 *(±26.6)*	5.85 *(±1.35)*	7.2 *(±0.08)*	5.97 *(±0.13)*	14.2 *(±3.1)*	0.38 *(±0.07)*
SSN2	2.48 *(±0.05)*	0.17 *(±0)*	177.91 *(±9.13)*	7.2 *(±0.4)*	7.3 *(±0.18)*	6.32 *(±0.25)*	18.4 *(±0.7)*	0.27 *(±0.01)*
SSN3	2.4 *(±0.11)*	0.22 *(±0.01)*	139.69 *(±6.94)*	8.21 *(±0.12)*	8.0 *(±0.07)*	6.7 *(±0.06)*	17.8 *(±1.9)*	0.22 *(±0.01)*

**(std error of the means)*
